# Post-traumatic endophthalmitis with Moraxella in a child

**DOI:** 10.3205/oc000174

**Published:** 2021-01-28

**Authors:** Upma Awasthi, Rohini Grover, Chetan Videkar, Abhishek Varshney

**Affiliations:** 1 Department of Vitreoretina, C. L. Gupta Eye Institute, Uttar Pradesh, India

**Keywords:** post-traumatic endophthalmitis, Moraxella sp., pediatric, pars plana vitrectomy, trauma, vancomycin

## Abstract

**Purpose:** To describe a case of post-traumatic endophthalmitis with *Moraxella* in a child.

**Methods:** Case report of an 11-year-old boy who presented with redness and profound visual loss in his left eye for 3 days following trauma with a sewing needle. Detailed ophthalmic examination showed hand movement vision, corneal edema with mobile hypopyon, as well as dot and clump echoes in Ultrasound B-scan. The clinical diagnosis of acute post-traumatic endophthalmitis was made.

**Result:** The patient underwent pars plana vitrectomy and vitreous biopsy, and was given intravitreal antibiotics (vancomycin 1 mg/0.1 ml, ceftazidime 2.25 mg/0.1 ml, voriconazole 0.1 mg/0.1 ml). Non-pigmented small colonies growth was observed on culture plates which were identified as *Moraxella*.

**Conclusion:** To date, no case report has been published regarding post-traumatic endophthalmitis due to *Moraxella* species in the pediatric age group.

## Introduction

Post-traumatic endophthalmitis has a poor prognosis, as described in the Ocular Trauma Score (OTS) [1]. Incidence of post-traumatic endophthalmitis in the <18 years age group ranges from 2.8% to 58% [2]. Duration of more than 24 hours, retained intraocular foreign body (IOFB), ruptured lens capsule or prolapsed ocular tissue and soil contamination are associated with an increased risk of endophthalmitis post-trauma [3], [4].

Causative organisms vary according to the mode of injury and age of the patient. In the pediatric population, the *Streptococcus* species are the most frequent causative organisms [4]. Only few reports on post-traumatic endophthalmitis caused by *Moraxella* have been published previously [5], [6], [7]. All of them were reported in the adult population [5], [6], [7]. This report is a case of post-traumatic endophthalmitis caused by the *Moraxella* species in an 11-year-old boy.

## Case description

**Figure 1:**
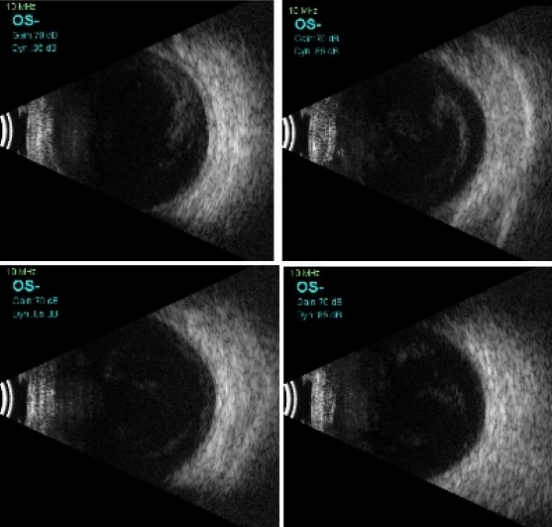
Transverse B-scan of all quadrants showing medium reflective dot and clump echoes in all quadrants in vitreous cavity

**Figure 2:**
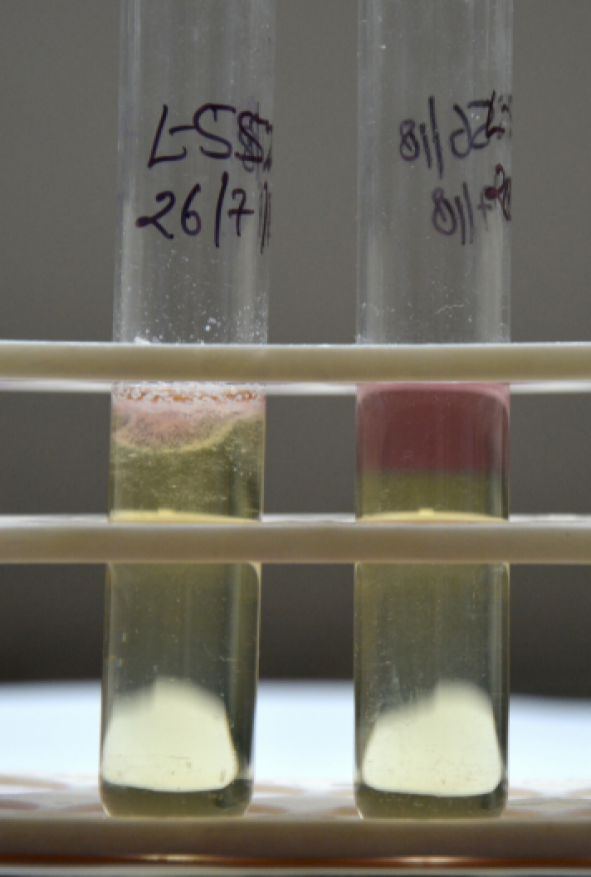
Brain heart infusion broth (BHIB) showing turbidity near the surface compared to the control medium; note that Moraxella is an aerobic bacteria thus growth is near the surface.

**Figure 3:**
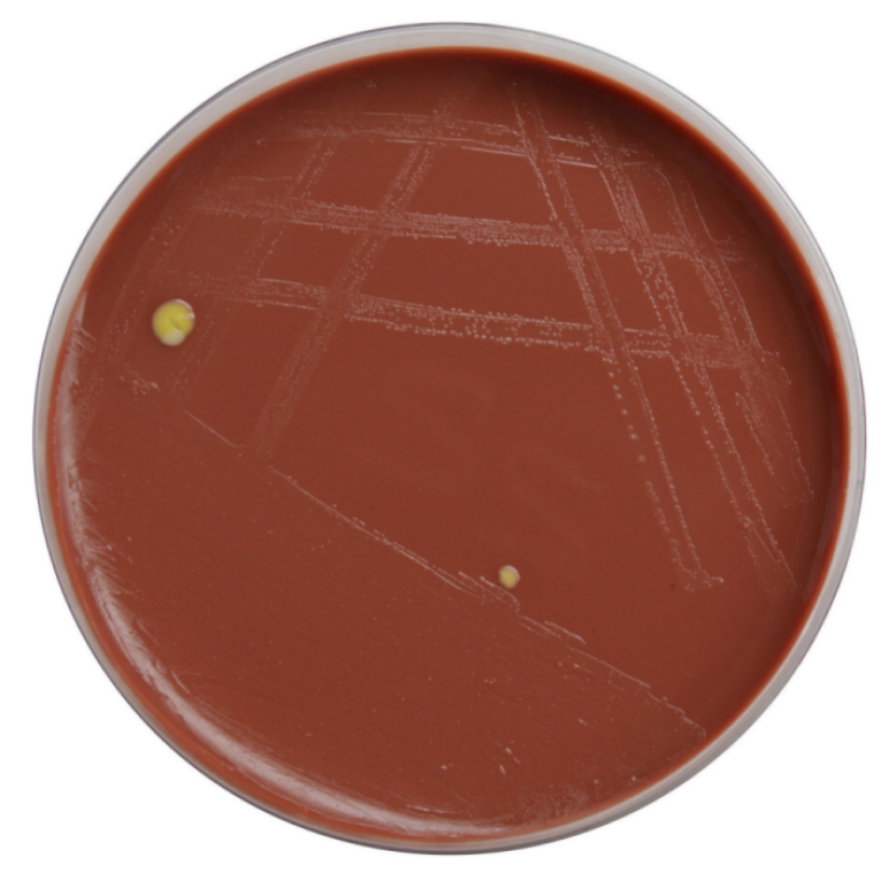
Growth on blood agar showing translucent, small 1 mm colonies with waxy surfaces

**Figure 4:**
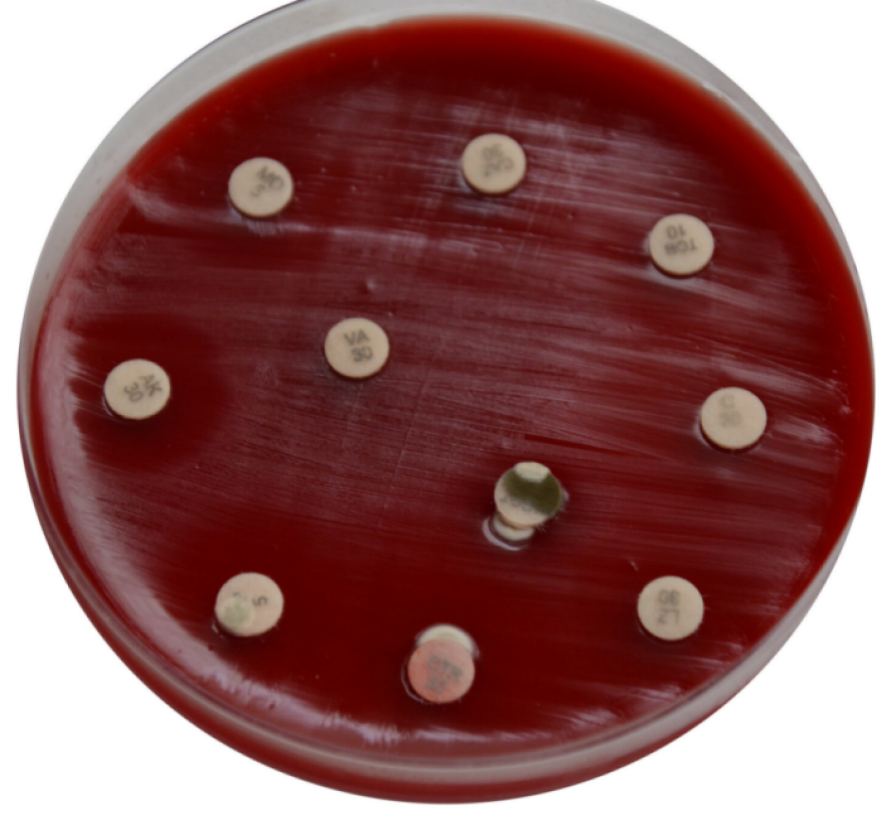
Antibiotic sensitivity of Moraxella for amikacin, ceftriaxone, ceftazidime, chloramphenicol, tobramycin, vancomycin and linezolid on blood agar with the Kirby-Bauer disc diffusion method

An 11-year-old boy presented to us with complaints of sudden decrease of vision, redness and white discoloration of the cornea in the left eye for the previous three days. The patient gave history of injury with a needle in his left eye. On examination, his best corrected visual acuity (BCVA) was 20/20 in the right eye and hand movement (HM) in the left eye. Intraocular pressure (IOP) with Goldmann applanation tonometry was 18 mm of Hg in both eyes. Right-eye examination was unremarkable. In the left eye, the lids were edematous, the conjunctiva was congested, the cornea had stromal edema and SPKs. 1 mm mobile hypopyon with fibrin was also present in the patient’s left eye. There was no view of fundus. Ultrasound B-scan revealed moderate numbers of moderate reflective dot and clump echoes in all quadrants (Figure 1 (Fig. 1)). The clinical diagnosis of post-traumatic endophthalmitis was made. The patient underwent pars plana vitrectomy and vitreous biopsy, and was given intravitreal antibiotics (vancomycin 1 mg/0.1 ml, ceftazidime 2.25 mg/0.1 ml, voriconazole 0.1 mg/0.1 ml). Vitreous aspirates were inoculated on chocolate agar (CA), blood agar (BA), thioglycolate broth, Sabaroud’s dextrose agar, and brain heart infusion broth (BHIB), and were sent for Gram and KOH stain. Direct mount on Gram stain showed Gram-variable cocci with plenty of polymorphonuclear cells in oil immersion field. On the 3^rd^ day, significant growth was observed on BA and CA, and turbidity was found in BHIB (Figure 2 (Fig. 2)). Colonies on BA and CA were translucent, small, easy to crumble, and had a waxy surface (Figure 3 (Fig. 3)). The isolate was identified as genus *Moraxella*, and subculture was sent for species identification with the VITEK 2 (version 5.02) system (BioMerieux, USA). However, the species was not determined by the VITEK 2 system. Antibiotic sensitivity was determined by the Kirby-Bauer disc diffusion method on blood agar. The isolate was found to be sensitive for amikacin, ceftriaxone, ceftazidime, chloramphenicol, and tobramycin, and was resistant to vancomycin and linezolid (Figure 4 (Fig. 4)). Medications were changed as per the culture and sensitivity reports. Intravitreal ceftazidime (2.25 mg/0.1 ml) and dexamethasone (0.4 mg/0.1 ml) was given. The patient responded well to the treatment, and his BCVA improved to 20/25 at two weeks follow-up. Thereafter, topical antibiotics were reduced in frequency. At 1 month follow-up, the patient’s visual acuity was 20/20 with no complaints.

## Discussion

*Moraxella* are Gram-negative, unstable organisms which are usually slow in decolorizing, thus are Gram-variable as in our case. They do not have flagellar motility and need high relative humidity for growth. Growth can be improved by the addition of blood, serum, or ascitic fluid [8].

The *Moraxella* species has been isolated from cases of chronic bacterial conjunctivitis and angular blepharitis, as its principal habitat are conjunctiva and nasal cavity [8]. *Moraxella* has been reported to cause delayed-onset endophthalmitis in patients with thin or leaky blebs [9].

There are three case reports of post-traumatic endophthalmitis caused by the *Moraxella* species [5], [6], [7]. The first case report was of an elderly male patient who developed endophthalmitis with trivial trauma with a contact lens [5]. The authors postulated that marked invasiveness of bacteria was due to the immunocompromised status of their patient [5]. In our case also, bacteria gained entry with trivial trauma with a needle, and our patient was neither immunocompromised nor elderly. We suspect that the direct penetration of the needle in the vitreous was the reason why a trial trauma led to endophthalmitis even in an immunocompetent patient. In their study on bleb-related endophthalmitis, Laukeland et al. reported a poor correlation between the conjunctiva and vitreous culture reports, and postulated that bacteria could only be present transiently on the bleb [9].

Two other patients had open-globe injuries and presented with poor visual acuity [6]. The final visual outcome was NPL (no perception of light) in both cases [6]. Our case achieved a final visual acuity of 20/20, though the presenting visual acuity was HM. As per OTS, our patient had a 44% chance of getting a final visual acuity better than 20/40 [1]. Endophthalmitis with *Moraxella* is reported to have better visual outcomes if there are no other comorbidities [6]. However, good visual outcomes have never been reported in post-traumatic cases. The good visual outcome in our case may be due to the early presentation and the prompt diagnosis and management.

In our case, culture sensitivity reports showed resistance to vancomycin. It has been reported in previous studies that *Moraxella* species should be tested for β-lactamase production. These β-lactamase-producing strains will be resistant to vancomycin [6], [10]. *Moraxella* are fastidious bacteria, thus blood agar was used for sensitivity. Furthermore, species identification is difficult or not possible in many of the cases [5], [9]. In our case as well, the species was not identified on the VITEK 2 system. In cases of vancomycin-resistant endophthalmitis, rare infectious agents such as *Moraxella* should be kept in mind and should be tested for.

To the best of our knowledge, this is the first case report of post-traumatic endophthalmitis by the *Moraxella* species in the pediatric age group. We also report favorable visual outcome which was not reported in post-traumatic cases.

## Notes

### Competing interests

The authors declare that they have no competing interests.

### Acknowledgments

The authors would like to thank Dr. V. K. Goel and Dr. Divya Goel of M. M. Diagnostics, Moradabad, Uttar Pradesh, India, for their efforts for species identification with the VITEK 2 system. The authors also acknowledge Mr. Lokesh Chauhan for his technical support.

## References

[1] Kuhn F, Maisiak R, Mann L, Mester V, Morris R, Witherspoon CD (2002). The Ocular Trauma Score (OTS). Ophthalmol Clin North Am.

[2] Alfaro DV, Roth DB, Laughlin RM, Goyal M, Liggett PE (1995). Paediatric post-traumatic endophthalmitis. Br J Ophthalmol.

[3] Gokce G, Sobaci G, Ozgonul C (2015). Post-Traumatic Endophthalmitis: A Mini-Review. Semin Ophthalmol.

[4] Bhagat N, Nagori S, Zarbin M (2011). Post-traumatic Infectious Endophthalmitis. Surv Ophthalmol.

[5] Ebright JR, Lentino JR, Juni E (1982). Endophthalmitis caused by Moraxella nonliquefaciens. Am J Clin Pathol.

[6] Berrocal AM, Scott IU, Miller D, Flynn HW (2001). Endophthalmitis caused by Moraxella species. Am J Ophthalmol.

[7] Lieb DF, Scott IU, Flynn HW, Miller D, Feuer WJ (2003). Open globe injuries with positive intraocular cultures: factors influencing final visual acuity outcomes. Ophthalmology.

[8] van Bijsterveld OP (1973). Host-parasite relationship and taxonomic position of Moraxella and morphologically related organisms. Am J Ophthalmol.

[9] Laukeland H, Bergh K, Bevanger L (2002). Posttrabeculectomy endophthalmitis caused by Moraxella nonliquefaciens. J Clin Microbiol.

[10] Sherman MD, York M, Irvine AR, Langer P, Cevallos V, Whitcher JP (1993). Endophthalmitis caused by beta-lactamase-positive Moraxella nonliquefaciens. Am J Ophthalmol.

